# Altered expression of ligands for the NKG2D and DNAM-1 activating receptors during HIV-1 infection

**DOI:** 10.1186/1742-4690-10-S1-P28

**Published:** 2013-09-19

**Authors:** Lia Vassena, Erica Giuliani, Giulia Matusali, Margherita Doria

**Affiliations:** 1 Bambino Gesù Children’s Hospital, Rome, Italy

## Background

Human cells may respond to viral infection or other stress by expressing on their membrane ligands for activating receptors present on cytotoxic NK and T cells, such as NKG2D and DNAM-1 receptors, thus eliciting recognition and elimination by the immune system. Work from our laboratory has shown that, upon infection with HIV-1, CD4^+^ T cells over-express ligands for NKG2D (MICA/B and ULBP2) and DNAM-1 (PVR), hence becoming susceptible to NKG2D-and DNAM-1-mediated lysis by NK cells. The cell-surface expression of activating ligands, however, is down-regulated by the HIV-1 Nef and Vpu proteins, a phenomenon that protects infected cells from cytotoxic responses to some extent. Here we further investigated the dual regulation of activating ligands by HIV-1 focusing on viral and cellular factors involved in up-regulation of ligands expression and on the capacity of HIV-infected cells to release soluble ligands in the extracellular environment.

## Materials and methods

Jurkat T cells and primary CD4+ T lymphocytes were transduced with HIV-1 or individual viral genes (wt or mutated). Ligands expression was analyzed by measuring mRNA, cell-surface and total protein levels. Activation of the DNA Damage Response (DDR) pathway and cell cycle profile were also analyzed. Soluble ligands were measured by ELISA in the medium of *in vitro* infected cells as well as in the plasma of HIV-infected patients. The impact of soluble ligands on the expression and function of their cognate receptor was investigated by FACS-based immunofluorescence analysis and cytotoxicity assays.

## Results

Results showed that the HIV-1 Vpr protein increases cell-surface and total PVR levels acting at a post-transcriptional level. As reported previously for NKG2D ligands, PVR was up-regulated by Vpr via activation of the ATR kinase that triggers the DDR pathway and G_2_ arrest. Increased expression of PVR and NKG2D ligands correlated with their higher release in HIV-infected T cell cultures. Moreover, treatment-naïve HIV-infected patients displayed increased plasma levels of soluble MICA and ULBP2 and reduced NKG2D expression on NK and CD8+ T cells. However, uptake of antiretroviral therapy (ART) resulted in the drop of soluble NKG2D ligands and recovery of NKG2D expression. Finally, we found that NKG2D ligands in patients’ plasma down-regulated NKG2D on NK and CD8+ T cells and impaired NKG2D-mediated cytotoxicity of NK cells.

## Conclusions

Attempts to boost the DDR-mediated up-regulation of NKG2D and DNAM-1 ligands represent novel attractive approaches to improve recognition and elimination of HIV-infected cells by the immune system. By promoting the release of soluble ligands, HIV-1 may simultaneously attenuate their expression on infected cells and down-regulate cognate receptors on effector cells, thus subverting immuno-surveillance against HIV-1 and opportunistic infections, but ART has the potential to avoid such immune dysfunction.

